# Oral Metformin Treatment Prevents Enhanced Insulin Demand and Placental Dysfunction in the Pregnant Rat Fed a Fructose-Rich Diet

**DOI:** 10.5402/2012/757913

**Published:** 2012-08-16

**Authors:** Ana Alzamendi, Hector Del Zotto, Daniel Castrogiovanni, Jose Romero, Andres Giovambattista, Eduardo Spinedi

**Affiliations:** ^1^Neuroendocrine Unit, IMBICE (CONICET La Plata-CICPBA), P.O. Box 403, 1900 La Plata, Argentina; ^2^CENEXA (CONICET La Plata-UNLP), Argentina; ^3^Reproductive Endocrinology Section, Perinat, 1900 La Plata, Argentina

## Abstract

The intake of a fructose-rich diet (FRD) in the normal female rat induces features similar to those observed in the human metabolic syndrome phenotype. We studied the impact of FRD administration to mothers on pregnancy outcome. On gestational day (Gd) zero rats were assigned to either group: *ad libitum* drinking tap water alone (normal diet, ND) or containing fructose (10% w/vol; FRD) through pregnancy; all rats were fed a Purina chow diet *ad libitum* ND and FRD rats were daily cotreated or not with metformin (60 mg/Kg/day oral; ND + MF and FRD + MF) and submitted to a high glucose load test on Gd 14. Additionally, placentas from different groups were studied on Gd 20. Data indicated that: (1) although FRD rats well tolerated glucose overload, their circulating levels of insulin were significantly higher than in ND rats; (2) the mesometrial triangle blood vessel area was significantly lower in placentas from FRD than ND dams; (3) the detrimental effects of FRD administration to mothers were ameliorated by metformin cotreatment. Our study suggests that excessive intake of fructose during pregnancy enhanced the risk for developing gestational diabetes and subsequent preeclampsia, and that metformin prevented the poor pregnancy outcome induced by FRD.

## 1. Introduction

The prevalence of gestational diabetes mellitus (GDM) has significantly increased during the last decades [1], and offspring born to mothers with GDM present a significantly increased risk of developing obesity and type 2 diabetes mellitus (T2DM) [2, 3]. Maternal anthropometric factors (e.g., prepregnancy body weight (BW), adiposity weight gain during pregnancy) account for the variance in offspring BW and adiposity at birth [1, 4], and on other risk factors as well [5, 6]. However, maternal nutrition is a highly important conditioning factor of fetal growth. GDM is characterized by impaired glucose tolerance/decreased insulin sensitivity, and mothers with GDM have an increased risk of developing preeclampsia (PE) [7, 8]. Although the pathogenesis of PE still remains unclear, it is accepted that PE is an endothelial cell disorder associated with marked maternal and neonatal morbidity [9]. On the other hand, insulin effectiveness and angiogenesis [10–12] are key factors for the control of endothelial cell function. Consequently, the presence of decreased insulin sensitivity in the mother may enhance the defects in angiogenic function [13, 14], leading to an increased risk of PE.

Maternal nutritional disturbances during critical developmental periods [15–17] are known to raise the offspring's risk of developing obesity and metabolic disorders in adult life [17–19]. One component that has currently been modified in diets is carbohydrates, mainly sucrose and fructose. Although fructose lacks any short-term stimulatory effect on insulin and leptin production [20], excessive fructose intake through the diet without appropriate pancreatic and adiposity responses could lead to long-term detrimental effects on the regulation of energy metabolism. Although nowadays the total daily caloric intake by the individual has risen, the per capita fructose intake (namely, from high fructose corn syrup and, to a lesser extent, fruits and vegetables) drastically increased [20] from 64 to 81 g/day during the last decades. This behavioral change enhanced the prevalence of several metabolic disorders [21]. We recently reported that the intake of a fructose-rich diet (FRD) for a short period of time enhances oxidative stress [22] and induces several features of the human metabolic syndrome phenotype in normal [23, 24] rats. Moreover, overweight development resulted in the male offspring nursed by dams fed an FRD during the lactation period [17]. However, only few studies [25, 26] have been devoted to better examine the impact of FRD intake on the dam's physiology.

The objective of the present study was to explore the impact of the intake of FRD, combined or not with oral metformin (an enhancer of insulin sensitivity) treatment, on pregnancy outcome. For this aim we studied in dams fed an FRD, treated or not with metformin, metabolic responses to glucose overload (at middle-late pregnancy) and placental functionality (at late pregnancy). A group of dams with inhibited angiogenesis, an accepted model of rat preeclampsia [27], was run in parallel.

## 2. Materials and Methods

### 2.1. Animals and Experimental Designs

Randomly cycling virgin adult female rats (Sprague-Dawley) were individually caged and allowed to mate for 24 hours. Rats displaying spermatozoa at their vaginal smears were considered pregnant (gestational day zero). Animals were employed and experimented by following protocols for animal use from the NIH guidelines for care and use of experimental animals. All experiments received approval from our Institutional Committee on Animal Experimentation. Pregnant rats were randomly allocated in five experimental groups: (a) *ad libitum *fed with Purina rat chow and tap water for drinking (normal diet; ND); (b) *ad libitum *fed with Purina rat chow and tap water containing D(−) Fructose (Biopack, Argentina, 10% w/v) in the drinking water (fructose rich diet; FRD) [17]; (c) similar to ND rats but orally co-treated with metformin (MF; Craveri Lab., Argentina; at the dose 50 mg/Kg/day [28], dissolved in the drinking water, ND + MF dams); (d) similar to FRD rats and co-treated with MF as mentioned above (FRD + MF); (e) additionally, for comparison purposes only, a group of mothers fed an ND were treated i.p., on gestational day (Gd) 5, with a general inhibitor of angiogenesis (suramin, Alexis Biochemicals, Enzo Life Sciences, USA, at a 100 mg/Kg dose; SUR + ND dams) [27].

#### 2.1.1. i.v. Glucose Tolerance Test (GTT) at Middle-Late Pregnancy (Experiment 1) 

On the morning of Gd 14, dams from different experimental groups were lightly anesthetized with ketamine and submitted to an i.v. (through the right jugular vein) high glucose load test (2 g glucose per Kg/BW) [17]. Blood samples were withdrawn before (sample time zero) and several times (5, 15, 30, 45, and 60 minutes) after glucose administration. Plasma glucose concentrations were determined by a commercial (Wiener Lab., Argentina) enzymatic-colorimetric assay. The circulating concentrations of insulin were determined by a specific radioimmunoassay previously described [17].

#### 2.1.2. Analysis of Placental Function at Late Pregnancy (Experiment 2)

 In this experiment, additional groups (ND, FRD, ND + MF, FRD + MF, and SUR + ND) of dams were studied. On the morning of Gd 20, rats were weighed and submitted to laparatomy (under light ketamine anesthesia). Viable fetuses were counted, removed, and weighed. Placentas were immediately dissected from random positions on each side-horn, weighed, and fixed for histological studies as described by Nash et al. [27].

### 2.2. Histological Studies in Placental Tissues

Placentas were dehydrated in graded ethanol series and embedded in paraffin according to a standard protocol. The entire placenta was sectioned into 7 mm thick sections, mounted on glass slides, and stained with hematoxylin-eosin and periodic acid-schiff (PAS). PAS staining visualizes structures with a high proportion of carbohydrate components (i.e., glycogen, glycoprotein, and proteoglycans) such as connective tissues, mucus, and basal laminae. Hence, it facilitates the identification of maternal vessels in the mesometrial triangle. In the PAS-stained placenta, randomly chosen slides were used for stereological evaluation of the area occupied by vessels compared with the total area of the mesometrial triangle. Morphometric measurements were performed using a Carl Zeiss microscope, a color monitor, and optimas software. The slides were scanned at a magnification of 40x and the total area of maternal vessel volume was measured on square fields running through the section and completely covering the mesometrial triangle area. Vessel area (in percentage of the total area) was used to estimate vessel volume in each experimental group. One operator performed all the measurements using a double-blind system.

### 2.3. Statistical Analysis

Data (expressed as means ± SEM) were analyzed by ANOVA either two-factor (diet-treatment) or with repeated measures, followed by post hoc comparisons with Fisher's test [24].

## 3. Results

### 3.1. Effect of Fructose Rich Diet Intake and Metformin Treatment on i.v. High Glucose Load Test in 14-Day-Pregnant Rats

The aim of Experiment 1 was focused on revealing whether the intake of an FRD, through the first 14 days of gestation, is a modifying factor of metabolic responses to glucose overload. While dams' body weight values and body weight gain did not differ among groups on both Gd 0 and Gd 14 (Table 1), the 14-day average of total calorie intake was slightly (*P* < 0.05 versus ND values) enhanced in FRD mothers, regardless of MF co-treatment (Table 2).

**Figure 1:**
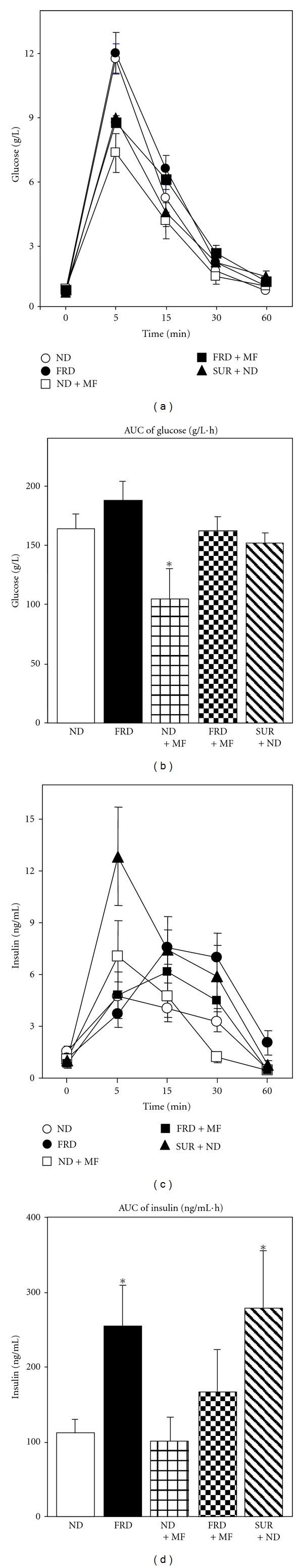
Circulating glucose (d) and insulin (c) levels before (sample time zero), and several times after i.v. glucose (2 g/Kg BW) administration in 14-day-pregnant ND (open circles), FRD (closed circles), MF + ND (open squares), MF + FRD (closed squares), and ND + SUR (closed triangles) rats. The areas under the curves (AUC) of plasma glucose (b) and insulin (d) concentrations throughout the test are also displayed. (Values are means ± SEM, *n* = 6/7 dams per group). *P < 0.05 versus ND.

Metabolic data indicated that on Gd 14, the nonfasting (basal, time zero values) peripheral levels of glucose and insulin were similar in all groups examined (Figures 1(a) and 1(c)). The profile of circulating glucose concentrations during the whole test showed that peak values occurred 5 min after glucose load in all groups examined (*P* < 0.05 versus respective basal values). While in FRD, FRD + MF, and SUR + ND dams 60 min plasma glucose levels remained significantly (*P* < 0.05) higher than the respective basal values; conversely, in ND and ND + MF rats those values were back to their respective basal values (Figure 1(a)). The analysis of the areas under the curve (AUC) of glucose values indicated that a significant (*P* < 0.05 versus ND values) reduction in this parameter accounted only in ND + MF dams (Figure 1(b)). Regarding plasma insulin levels, while in ND, ND + MF and SUR + ND groups peak plasma levels occurred 5 min after glucose load, such a parameter was delayed upto time 15 min in FRD and FRD + MF dams (Figure 1(c)) On time 60 min after glucose, circulating insulin concentrations were either back to (FRD, FRD + MF, and SUR + ND) or even lower than (ND, ND + MF) their respective basal values (Figure 1(c)). It should be noted, however, that on this time, plasma insulinemia was significantly (*P* < 0.05) higher in FRD than in ND dams. These differences can be clearly observed when the AUCs of plasma insulin concentrations are examined. In fact, the AUC of circulating insulin values were significantly (*P* < 0.05) higher in FRD than in ND dams (Figure 1(d)). Interestingly, MF treatment in dams fed an FRD partially prevented the enhanced insulin demand induced by the high glucose-load, although MF by itself did not modify overall insulin secretion in ND dams (Figure 1(d)). Finally, the peripheral parameters analyzed in SUR + ND dams indicated that they normally managed glucose homeostasis (Figures 1(a) and 1(b)), but it occurred at the expense of enhanced glucose-stimulated insulin release (Figures 1(c) and 1(d)).

### 3.2. Impact of Fructose Rich Diet Intake Alone or Combined with Metformin Treatment on Anthropometric Characteristics and Pregnancy Outcome on Gestational Day 20

Changes in body characteristics (BW and weight gain; Table 1) and total caloric intake (Table 2) on Gd 20 were quite similar to those found on Gd 14 (see also Tables 1 and 2). After laparatomy, we found that litter size and placental tissue weight did not differ among groups (Table 3). Conversely, fetuses from FRD dams were significantly (*P* < 0.05 versus ND) heavier than the normal ones, even when as a ratio with the corresponding placental weight (Table 3). MF treatment in ND dams rendered lighter (*P* < 0.05) fetuses than those recorded in ND mothers (Table 3), although this difference no longer exists after being expressed in relation to the corresponding placental weight (Table 3). Interestingly, the enhancement in fetuses BW (and in the relation between individual BW and placental weight) induced by FRD intake was fully prevented by co-treating FRD dams with MF (Table 3). Angiogenesis-inhibited dams displayed light fetuses (Table 3). Finally, means of the number of reabsorptions were similar in all groups (ranging between 0.32 and 0.93 re-absorptions per dam; *n* = 10/14 mothers group).

### 3.3. Fructose Rich Diet Intake-Induced Placental Dysfunction in the 20-Day-Pregnant Rat: Beneficial Effect of Metformin Treatment

**Figure 2:**
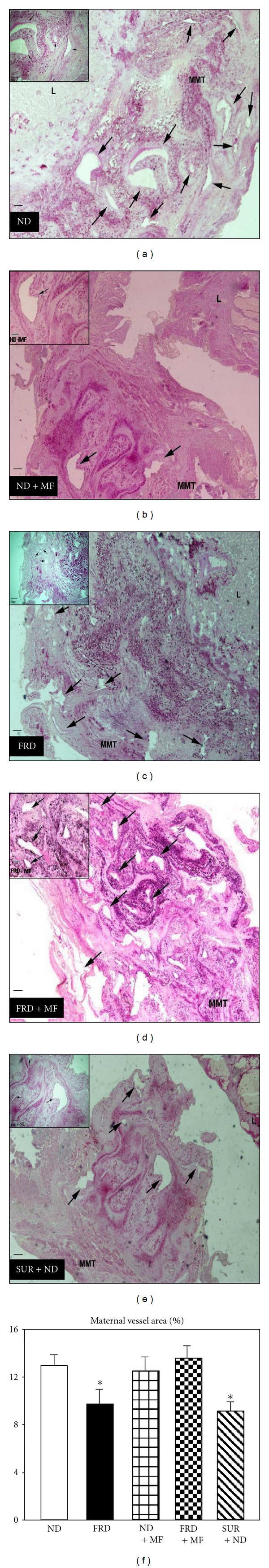
Representative placental mesometrial triangle (MMT) area of implanted offspring in 20-day-pregnant ND (a), FRD (c), MF + ND (b), MF + FRD (d), and ND + SUR (e) rats stained with PAS (L: labyrinth; Bars: 200 *μ*m). Arrows show vessel area (×4). Insets: corresponding MMT area at a higher magnification (10x). Maternal vessel area (in percentage) on gestational day 20 in the different groups of dams (f; values are means ± SEM, *n* = 9 specimens per group). *P < 0.05 versus ND values.

Then we examined the effects of FRD intake through 20 days of the gestational period, on placental functionality (Experiment 2). Placentas obtained of mothers from all groups on Gd 20 displayed a similar general morphology, with a large labyrinth with an adjacent trophospongium area (Figure 2, panels a–e). The mesometrial triangle was filled with decidual tissue and contained numerous PAS-positive arteries with endotrophoblast invasion. The total vessel area in placentas from FRD dams was significantly (*P* < 0.05) lower than that measured in ND rats (Figure 2(f)). Interestingly, in mothers fed an FRD and co-treated with MF, total blood vessel area was normally preserved (Figure 2(f)); thus indicating the effectiveness of MF treatment on FRD intake-induced placental dysfunction. Conversely, this parameter remained unaltered in dams fed an ND and co-treated with MF (see also Figure 2(f)). Accordingly to a previous study [27], the total placental blood vessel area in SUR + ND dams resulted also significantly (*P* < 0.05 versus ND group values) diminished, reaching a similar percentage of that recorded in FRD specimens (Figure 2(f)).

## 4. Discussion

The current data suggest that rats fed an FRD through pregnancy are at a high risk for developing GDM and subsequent PE. This notion is supported by two key findings: (a) an early (Gd 14) enhancement in high-glucose load-induced insulin secretion in plasma; (b) a distorted placental function observed at a late stage (Gd 20) of pregnancy. Highly important is the finding that these deleterious effects of FRD administration to dams was overridden by co-treatment with a low oral metformin dose.

We have previously addressed that nonpregnant rats fed an FRD during 3 weeks, developed high plasma levels of fatty acids, triglycerides, and inflammatory adipokines, as well as distorted adipose tissue morphology and function [24], with being these metabolic-endocrine abnormalities being common to those ascribed to the human metabolic syndrome phenotype [29]. In the present study we extended the information regarding the detrimental effects of FRD administered to pregnant rats. Of relevance is that the undesirable effects induced by the FRD were fully overridden by metformin. Indeed, as observed in non-pregnant normal rats [24], an enhanced insulin secretion in plasma occurred after glucose overload in FRD dams, thus clearly indicating that pregnant FRD rats developed substantial changes in peripheral insulin sensitivity, being the pancreatic reserve still capable for maintaining glucose homeostasis. This fact put in evidence the severity of the *allostatic* condition (FRD) during middle-late (Gd 14) pregnancy, and it is indicative for, although incomplete (e.g., FRD dams did not display any evidence of intolerance to glucose overload), the development of GDM. GDM incidence directly correlates with maternal body weight, in both human [30] and rodents [31]; however, we found that even at a normal body weight range, FRD dams are at a higher risk of GDM. Moreover, we found that treating dams fed an FRD with metformin, the enhanced demand in insulin release after high glucose load was completely abolished, indicating that MF is a highly effective treatment for assuring a normal pancreatic *β*-cell response in mothers at a high risk of GDM. In this regard, recent studies in pregnant women with GDM indicate that MF treatment effectively ameliorates pregnancy outcome [32]. In addition, we now provide data regarding the effect of an early inhibition of angiogenesis on carbohydrate metabolism in dams. In fact, suramin-treated mothers displayed an increased insulin demand in order to maintain glucose homeostasis during the high glucoseload test, thus clearly indicating that a diminished tissue blood supply has taken place in these dams.

As occurring in humans, FRD mothers were at a high risk for subsequent development of PE. Indeed, on Gd 20, we observed that FRD dams displayed a significantly reduced placental blood vessel area, parameter that was at a similar level in suramin-treated dams, an openly accepted rat model for rat PE [27]. It is plausible to expect that FRD-induced impaired insulin sensitivity could be a modifying factor of placental angiogenesis, due to the well-known pro-angiogenic effect of insulin [10–14] and its inhibitory activity on cell ephrin-B2 gene expression [33]. Thus, it could be expected that the impaired insulin sensitivity installed in FRD dams is responsible, at least in part, for reduced placental blood vessel area. This presumption finds support in the similarity of placental data obtained in suramin-treated and FRD dams. Indeed, it has been showed that suramin treatment in dams drastically reduced placental and renal blood vessel areas, being these parameters indicative for the hypertensive state developed by these dams [34]. Interestingly, and in accordance with its improving effect on glucose homeostasis, metformin treatment in FRD dams fully prevented placental dysfunction.

Finally and important to remark is the fact that the main index of abnormal pregnancy outcome that we found in FRD dams was the enhanced fetuses' body weight on Gd 20, regardless of any dam's overweight. It is accepted that during GDM, the compensatory mother's hyperinsulinemia is the most important signal causing macrosomic offspring [35]. Despite the placental dysfunction found in our FRD mothers, the enhanced insulin secretion operating for the maintenance of glucose homeostasis could be cooperating for the enhanced fetuses' body weight [35], an abnormality overridden by metformin treatment in FRD dams. In this regard, it is known that GDM directly correlates with high body weight of newborns [36]. It has been reported that when GDM women are treated with metformin, a compound readily crossing the placenta [37], the incidence of both macrosomic rate and large for gestational age neonates is reduced [38].

In conclusion, our data strongly suggest that the intake of an FRD during pregnancy induced early (Gd 14) changes in peripheral insulin sensitivity, that later (Gd 20) results in a high risk for the development of placental dysfunction and litter morbidity. Of relevance, the oral co-treatment with metformin counteracted the undesirable effects induced by the intake of FRD through pregnancy. It remains to be examined whether neonates born to FRD mothers are at high risk for further development of type 2 DM and/or obesity, and the effectiveness of metformin treatment in FRD dams on those litter's risks. 

## Figures and Tables

**Table 1:** Body weight values (BW, in grams) of dams, recorded on gestational day (Gd) zero, fourteen, and twenty. The percent of increase in BWs (in accordance with corresponding BWs on Gd 0) on Gd 14 and Gd 20 is also depicted. Values are means ± SEM (*n* = 14/16 rats on Gd 0; *n* = 7/8 rats on Gd 14 and Gd 20).

	BW Gd 0	BW Gd 14	% increase Gd 14	BW Gd 20	% increase Gd 20
ND	246.2 ± 9.9	286.5 ± 9.6	16.8 ± 2.9	342.5 ± 13.9	38.9 ± 3.5
FRD	241.1 ± 6.1	286.9 ± 6.8	19.9 ± 2.1	346.6 ± 11.7	43.2 ± 2.1
ND + MF	245.9 ± 7.2	286.8 ± 6.2	16.9 ± 2.5	345.5 ± 14.4	40.3 ± 2.4
FRD + MF	228.8 ± 5.7	273.8 ± 3.3	19.9 ± 2.6	338.9 ± 9.3	44.1 ± 5.1
SUR + ND	244.9 ± 5.9	273.6 ± 6.7	14.2 ± 1.9	326.8 ± 12.9	33.4 ± 3.5

**Table 2:** Average of daily energy intake (in cal per day, expressed as total and accordingly to the source derived from: food and fluid) for both 1–14- and 1–20-day periods in different experimental groups of dams. Values are means ± SEM (*n* = 14/16 rats on Gd 0; *n* = 7/8 rats on Gd 14 and Gd 20).

		14-day energy intake	20-day energy intake
	Total	91.1 ± 3.9	101.8 ± 8.1
ND	Food	91.1 ± 3.9	101.8 ± 8.1
	Fluid	—	—

	Total	114.9 ± 2.6^∗^	123.7 ± 4.3^∗^
FRD	Food	84.7 ± 3.2	87.2 ± 3.7
	Fluid	31.4 ± 2.6	34.1 ± 3.1

	Total	97.2 ± 4.1	103.7 ± 5.4
ND + MF	Food	97.2 ± 4.1	103.7 ± 5.4
	Fluid	—	—

	Total	109.1 ± 3.3^+^	129.3 ± 2.8^+^
FRD + MF	Food	79.5 ± 4.1	82.5 ± 8.9
	Fluid	29.6 ± 2.4	30.6 ± 4.3

	Total	86.9 ± 4.2	96.3 ± 4.7
SUR + ND	Food	86.9 ± 4.2	96.3 ± 4.7
	Fluid	—	—

*P < 0.05 versus ND values.

^+^ *P* < 0.05 versus MF + ND values.

**Table 3:** Litter size (*n* = 10–14 mothers per group), fetuses' body weight (*n* = 46–70 fetuses from 6-7 mothers per group) and placenta weight (*n* = 12–20) obtained from different groups of mothers on Gd 20. Values means ± SEM.

	Litter size	Fetuses BW (grams)	Placenta weight (grams)	Fetuses BW: placental weight
ND	11.3 ± 0.51	3.49 ± 0.05	0.68 ± 0.05	4.73 ± 0.32
FRD	11.4 ± 0.62	3.68 ± 0.04^∗^	0.75 ± 0.08	6.39 ± 0.39^∗^
ND + MF	11.2 ± 0.53	3.36 ± 0.05^∗^	0.71 ± 0.02	4.85 ± 0.42
FRD + MF	10.2 ± 0.86	3.33 ± 0.03^∗+^	0.78 ± 0.06	4.65 ± 0.38^+^
SUR + ND	10.3 ± 0.67	3.35 ± 0.04^∗^	0.66 ± 0.05	4.97 ± 0.46

*P < 0.05 versus ND values.

^+^ *P* < 0.05 versus FRD values.

## References

[1] Catalano PM, Kirwan JP, Haugel-De SM, King J (2003). Gestational diabetes and insulin resistance: role in short- and long-term implications for mother and fetus. *Journal of Nutrition*.

[2] Silverman BL, Rizzo TA, Cho NH, Metzger BE (1998). Long-term effects of the intrauterine environment: the northwestern university diabetes in pregnancy center. *Diabetes Care*.

[3] Pettitt DJ, Nelson RG, Saad MF, Bennett PH, Knowler WC (1993). Diabetes and obesity in the offspring of Pima Indian women with diabetes during pregnancy. *Diabetes Care*.

[4] Vohr BR, McGarvey ST, Tucker R (1999). Effects of maternal gestational diabetes on offspring adiposity at 4–7 years of age. *Diabetes Care*.

[5] Kvehaugen AS, Andersen LF, Staff AC (2010). Anthropometry and cardiovascular risk factors in women and offspring after pregnancies complicated by preeclampsia or diabetes mellitus. *Acta Obstetricia et Gynecologica Scandinavica*.

[6] Bernstein IM, Catalano PM (1994). Examination of factors contributing to the risk of cesarean delivery in women with gestational diabetes. *Obstetrics and Gynecology*.

[7] Schmidt MI, Duncan BB, Reichelt AJ (2001). Gestational diabetes mellitus diagnosed with a 2-h 75-g oral glucose tolerance test and adverse pregnancy outcomes. *Diabetes Care*.

[8] Wolf M, Sandler L, Muñoz K, Hsu K, Ecker JL, Thadhani R (2002). First trimester insulin resistance and subsequent preeclampsia: a prospective study. *Journal of Clinical Endocrinology and Metabolism*.

[9] Thadhani R, Ecker JL, Mutter WP (2004). Insulin resistance and alterations in angiogenesis: additive insults that may lead to preeclampsia. *Hypertension*.

[10] Chou E, Suzuma I, Way KJ (2002). Decreased cardiac expression of vascular endothelial growth factor and its receptors in insulin-resistant and diabetic states: a possible explanation for impaired collateral formation in cardiac tissue. *Circulation*.

[11] Montagnani M, Ravichandran LV, Chen H, Esposito DL, Quon MJ (2002). Insulin receptor substrate-1 and phosphoinositide-dependent kinase-1 are required for insulin-stimulated production of nitric oxide in endothelial cells. *Molecular Endocrinology*.

[12] Vicent D, Ilany J, Kondo T (2003). The role of endothelial insulin signaling in the regulation of vascular tone and insulin resistance. *Journal of Clinical Investigation*.

[13] Luft FC (2006). Soluble endoglin (sEng) joins the soluble fms-like tyrosine kinase (sFlt) receptor as a pre-eclampsia molecule. *Nephrology Dialysis Transplantation*.

[14] Kappas NC, Zeng G, Chappell JC (2008). The VEGF receptor Flt-1 spatially modulates Flk-1 signaling and blood vessel branching. *Journal of Cell Biology*.

[15] Hamilton JK, Odrobina E, Yin J, Hanley AJ, Zinman B, Retnakaran R (2010). Maternal insulin sensitivity during pregnancy predicts infant weight gain and adiposity at 1 year of age. *Obesity*.

[16] Patel MS, Srinivasan M (2010). Metabolic programming due to alterations in nutrition in the immediate postnatal period. *Journal of Nutrition*.

[17] Alzamendi A, Castrogiovanni D, Gaillard RC, Spinedi E, Giovambattista A (2010). Increased male offspring’s risk of metabolic-neuroendocrine dysfunction and overweight after fructose-rich diet intake by the lactating mother. *Endocrinology*.

[18] Taylor PD, Poston L (2007). Developmental programming of obesity in mammals. *Experimental Physiology*.

[19] McMillen IC, Robinson JS (2005). Developmental origins of the metabolic syndrome: prediction, plasticity, and programming. *Physiological Reviews*.

[20] Elliott SS, Keim NL, Stern JS, Teff K, Havel PJ (2002). Fructose, weight gain, and the insulin resistance syndrome. *American Journal of Clinical Nutrition*.

[21] Bray GA, Nielsen SJ, Popkin BM (2004). Consumption of high-fructose corn syrup in beverages may play a role in the epidemic of obesity. *American Journal of Clinical Nutrition*.

[22] Rebolledo OR, Marra CA, Raschia A, Rodriguez S, Gagliardino JJ (2008). Abdominal adipose tissue: early metabolic dysfunction associated to insulin resistance and oxidative stress induced by an unbalanced diet. *Hormone and Metabolic Research*.

[23] Alzamendi A, Giovambattista A, Raschia A (2009). Fructose-rich diet-induced abdominal adipose tissue endocrine dysfunction in normal male rats. *Endocrine*.

[24] Alzamendi A, Castrogiovanni D, Ortega HH, Gaillard RC, Giovambattista A, Spinedi E (2010). Parametrial adipose tissue and metabolic dysfunctions induced by fructose-rich diet in normal and neonatal-androgenized adult female rats. *Obesity*.

[25] Koski KG, Fergusson MA (1992). Amniotic fluid composition responds to changes in maternal dietary carbohydrate and is related to metabolic status in term fetal rats. *Journal of Nutrition*.

[26] Rawana S, Clark K, Zhong S, Buison A, Chackunkal S, Jen KLC (1993). Low dose fructose ingestion during gestation and lactation affects carbohydrate metabolism in rat dams and their offspring. *Journal of Nutrition*.

[27] Nash P, Wentzel P, Lindeberg S (2005). Placental dysfunction in Suramin-treated rats—a new model for pre-eclampsia. *Placenta*.

[28] Yilmaz B, Sucak A, Kilic S (2010). Metformin regresses endometriotic implants in rats by improving implant levels of superoxide dismutase, vascular endothelial growth factor, tissue inhibitor of metalloproteinase-2, and matrix metalloproteinase-9. *American Journal of Obstetrics and Gynecology*.

[29] Bartha JL, González-Bugatto F, Fernández-Macías R, González-González NL, Comino-Delgado R, Hervías-Vivancos B (2008). Metabolic syndrome in normal and complicated pregnancies. *European Journal of Obstetrics Gynecology and Reproductive Biology*.

[30] Gibson KS, Waters TP, Catalano PM (2012). Maternal weight gain in women who develop gestational diabetes mellitus. *Obstetrics and Gynecology*.

[31] Holemans K, Caluwaerts S, Poston L, Van Assche FA (2004). Diet-induced obesity in the rat: a model for gestational diabetes mellitus. *American Journal of Obstetrics and Gynecology*.

[32] Silva JC, Fachin DR, Coral ML, Bertini AM (2012). . Perinatal impact of the use of metformin and glyburide for the treatment of gestational diabetes mellitus. *Journal of Perinatal Medicine*.

[33] Hiden U, Lang I, Ghaffari-Tabrizi N, Gauster M, Lang U, Desoye G (2009). Insulin action on the human placental endothelium in normal and diabetic pregnancy. *Current Vascular Pharmacology*.

[34] Nash P, Olovsson M, Eriksson UJ (2005). Placental dysfunction in Suramin-treated rats: impact of maternal diabetes and effects of antioxidative treatment. *Journal of the Society for Gynecologic Investigation*.

[35] Khan NA (2007). Role of lipids and fatty acids in macrosomic offspring of diabetic pregnancy. *Cell Biochemistry and Biophysics*.

[36] Lapolla A, Dalfrà MG, Ragazzi E, De Cata AP, Fedele D (2011). New international association of the diabetes and pregnancy study groups (IADPSG) recommendations for diagnosing gestational diabetes compared with former criteria: a retrospective study on pregnancy outcome. *Diabetic Medicine*.

[37] Charles B, Norris R, Xiao X, Hague W (2006). Population pharmacokinetics of metformin in late pregnancy. *Therapeutic Drug Monitoring*.

[38] Gandhi P, Bustani R, Madhuvrata P, Farrell T (2012). Introduction of metformin for gestational diabetes mellitus in clinical practice: has it had an impact?. *European Journal of Obstetrics & Gynecology and Reproductive Biology*.

